# Construction of decision tree prediction model and group intervention for the mindfulness level of community pre-frail elderly: A cross-sectional study

**DOI:** 10.1097/MD.0000000000044878

**Published:** 2025-10-03

**Authors:** Hao Zheng, Junrong Xu, Fei Li, Yi Qin, Daoxun Zheng

**Affiliations:** a Faculty of Nursing, Guangxi University of Chinese Medicine, Nanning, Guangxi, China; b Geriatric Department, People’s Hospital of Guigang, Guigang, Guangxi, China; c Faculty of Nursing, Guangxi Medical University, Nanning, Guangxi, China; d The First Affiliated Hospital, Guangxi University of Chinese Medicine, Nanning, Guangxi, China; e Department of Neurology, Nanyang Second People’s Hospital, Nanyang, Henan, China.

**Keywords:** mental health, mindfulness, older adults, pre-frailty, psychological stress

## Abstract

Pre-frailty, a reversible state before frailty, is key for intervention. Mindfulness, a protective factor against frailty, is underexplored in community-dwelling pre-frail older adults. This study identified its influences, built predictive models, and proposed stratified interventions. 156 pre-frail older adults meeting inclusion and exclusion criteria were recruited from 5 communities in Nanning City, Guigang City, and Yulin City between August 2024 and February 2025 via convenience sampling. Data were collected using a General Demographic Questionnaire, the Chinese Frailty Scale, and the Mindful Attention Awareness Scale (Chinese version). Univariate analysis and multiple linear stepwise regression were employed to identify influencing factors, and a decision tree algorithm was applied to construct the prediction model. The mean mindfulness score among community-dwelling pre-frail older adults was 70.46 ± 9.80 (95% CI: 68.91–72.01), indicating a moderate level. Multiple linear stepwise regression identified monthly income as the primary influencing factor (β = 0.208, *P* = .011). Secondary factors significantly associated with mindfulness levels were medical burden, place of residence, and insomnia, with no evidence of multicollinearity (*P* < .05, VIF < 5). The decision tree model comprised 4 layers, 11 nodes, and 6 terminal nodes. Monthly income emerged as the most significant predictor (root node *F* = 18.645, *P* < .001), using healthcare burden, insomnia status, number of children, and educational attainment as additional classification nodes. Both methods consistently verified monthly income as the primary predictor. Establishing a targeted intervention framework integrating “Economic Support–Sleep Management–Intergenerational Support” was recommended. prioritizing support for the low-income group with medical burdens through integrated measures including economic assistance, pain management, bundled medical-psychological services, and community care centers; promoting “sleep-mindfulness” combined interventions and adapting urban community support models to rural settings to bridge urban–rural gaps; These findings provide evidence-based guidance for enhancing mental health and delaying frailty progression in this population.

## 1. Introduction

In 2020, the global population aged 65 years and older reached 727 million. With accelerating population aging, the absolute number of older adults is projected to increase further. Approximately 16% of community-dwelling older adults worldwide are identified as frail, while multidimensional assessments indicate that about one-quarter exhibit frailty and one-third are in a pre-frail state.^[1,2]^ Frailty is an age-related clinical syndrome characterized by decreased physiological reserve and multisystem dysfunction, leading to increased vulnerability and diminished resistance to stressors.^[3,4]^ Its core manifestations can be assessed using the Chinese version of the FRAIL Scale, including involuntary weight loss, fatigue, decreased endurance, limited physical activity, and multimorbidity. Distinct from mere aging or comorbidity alone, frailty represents a dynamic process reflecting overall functional decline in the organism, which significantly increases the risks of falls, disability, hospitalization, and mortality.^[5,6]^ In intervention studies, pre-frailty refers to a transitional state where some frailty characteristics are present but the diagnostic criteria for full frailty are not yet met. This state represents a critical window for reversing functional decline.^[7,8]^

The mental health challenges among pre-frail older adults in community home-based settings have become increasingly prominent. Mindfulness Level, defined as an individual’s capacity for awareness and acceptance of present-moment experiences, has been established as a key protective factor in delaying the progression of frailty.^[9]^ Mindfulness refers to a form of awareness that arises through purposefully attending to present-moment experiences without judgment.^[10]^ As a method of focusing attention on the present, mindfulness enhances positive emotions in response to life events through active self-regulation, thereby improving physical and mental well-being.^[11,12]^ Mindfulness theory posits that high-level mindfulness may effectively mitigate the decline in physical and cognitive function among pre-frail older adults by improving emotion regulation, reducing stress reactivity, and promoting health behaviors.^[13]^

Current research provides limited support for the notion that frailty directly causes reduced mindfulness. Conversely, mindfulness levels show positive correlations with psychological well-being, resilience, and quality of life. Mindfulness-based interventions, such as yoga, Tai Chi, and meditation, have been shown to improve physical function, cognitive performance, emotional health, and quality of life in older adults, all of which are closely associated with frailty.^[14,15]^

Currently, mindfulness levels among older adults with chronic diseases in Chinese communities are generally low and closely associated with socioeconomic status, health conditions, and psychological burden.^[16]^ Lower mindfulness levels may impair physical and mental health, and cognitive function, exacerbate family conflicts, trigger psychological disorders, ultimately straining healthcare resources and undermining population health metrics. Limited research exists on developing stratified intervention approaches aligned with mindfulness-based risk characteristics. This study aims to integrate dual modeling approaches (multiple linear regression and CRT decision trees) to: Decipher interaction networks among economic-health-social factors, construct precision intervention models based on decision tree clustering, provide community practitioners with evidence-based strategies to support the global “healthy aging” initiative.^[17]^

Given that the mindfulness level of pre-frail older adults is affected by the complex interaction of economic, health, and social factors, this study employed a dual-model strategy combining multiple linear regression and decision tree: the Former quantifies the independent effects of influencing factors, while the latter parses the interaction pathways between factors. Together, they verify monthly income as the primary predictive factor, as well as medical burden and insomnia as 2 secondary factors, identify high-risk subgroups (e.g., the PCOA-1 group), achieve methodological complementarity, and provide a classification basis for precision intervention. When exploring the influencing factors of mindfulness level in community-dwelling pre-frail older adults, a single model can hardly fully capture the complex relationships between variables. Although stepwise multiple linear regression can quantify the independent effects of various factors, for example, identifying the net impact intensity of variables such as monthly income and medical burden, it is limited by the linear assumption and thus difficult to reveal the interactions between factors, for example, the synergistic impact of economic status and sleep problems. In contrast, while decision trees excel at presenting hierarchical interaction pathways of variables through recursive partitioning, they cannot quantify the weight of independent contributions of each factor.

Therefore, this study adopted a dual-model strategy: On the one hand, stepwise multiple linear regression was used to clarify the intensity of independent impacts of each factor on mindfulness level and the risk of multicollinearity, providing a basis for target priority for intervention; on the other hand, the Classification and Regression Tree (CRT) decision tree was employed to explore the nonlinear interaction relationships between factors and identify high-risk subgroups with homogeneous characteristics, such as the low-income and high medical burden combined group, offering pathway specificity references for grouped intervention. The complementarity of the 2 methods can simultaneously meet the research needs of quantifying effects and parsing mechanisms, thereby providing more systematic theoretical support for community-based precision intervention.

## 2. Materials and methods

### 2.1. Ethics statement

A cross-sectional study was conducted and approved by the Ethics Committee of Guangxi University of Chinese Medicine on March 1, 2024 (Ethic No. GXUCMIRBTM2024-02-118). All procedures strictly adhered to the principles of the Declaration of Helsinki. The research solely involved the collection of general demographic information and questionnaire data, posing relatively low risk to participants. All participants provided signed informed consent forms and retained the right to withdraw unconditionally at any stage.

### 2.2. Sample size calculation

The sample size calculation method for this study is 5 to 10 times the number of independent variables. In this study, there are 12 independent variables including age, sex, height, weight, education, monthly income, number of children’s residences, medical payment methods, healthcare burden, daily exercise duration, pain status and sleep quality. Considering a 20% dropout rate. Thus, the calculated sample size should be 144 cases.

### 2.3. Data sources

The study design of this study is cross-sectional study. From August 2024 to February 2025, a convenience sampling of 156 pre-frail community-dwelling older adults (PCOA) meeting inclusion criteria was recruited from 5 communities in Nanning, Guigang, and Yulin cities. Diagnostic criteria is defined by the Chinese version of the Frail Scale,^[18]^ which comprises 5 dichotomous items with score range 0 to 5.0 means non-frail, 1 to 2 score means pre-frail, score more than 3 means frail. Inclusion criteria includes: Frail score is 1 to 2^[18]^; Community-dwelling with age 60 years and above^[19]^; Provision of written informed consent; Clear consciousness with adequate literacy and verbal capacity for communication. Exclusion criteria includes: Severe physical and psychiatric disorders, Alzheimer disease, or cognitive impairment; Visual and hearing impairments hindering communication; Inability for self-care.

### 2.4. Measurement instruments

#### 2.4.1. Demographic questionnaire

Demographic questionnaire developed based on geriatric expert consultations and literature,^[20,21]^ covering: Sociodemographics (age, sex, height, weight, education, monthly income, residence), number of children, medical payment methods, healthcare burden, daily exercise duration, pain status (Visual Analogue Scale and Numerical Rating Scale implied), and sleep quality (Pittsburgh Sleep Quality Index).

#### 2.4.2. Frail frailty screening scale

This scale was developed by geriatric experts from the International Association of Nutrition and Aging (IANA) based on the frailty phenotype and frailty index.^[18]^ It comprises 5 items with a dichotomous response format (“Yes” or ‘No’), scored as 1 or 0 respectively, yielding a total score ranging from 0 to 5. A score more than 3 indicates frailty, 1 to 2 scores indicates pre-frailty, and 0 indicates non-frailty. The scale demonstrates adequate reliability and validity, with a Cronbach α coefficient of 0.826 and a Kaiser-Meyer-Olkin (KMO) value of 0.766.^[22,23]^

#### 2.4.3. Mindfulness attention awareness scale

This scale was developed by Brown and Ryan. It consists of 4 dimensions and 15 items, covering the domains of cognition, physiology, emotion, and sensation. A 6-point Likert scale is used for scoring. For each item, respondents choose from “always,” “often,” “sometimes,” “occasionally,” “rarely,” and “never,” which correspond to scores of 1, 2, 3, 4, 5, and 6 respectively. The total score ranges from 15 to 90. A total score of 66 to 90 indicates a high level of mindfulness, 41 to 65 indicates a moderate level, and 1 to 40 indicates a low level. The scale has a Cronbach ɑ coefficient of 0.89, a test-retest reliability of 0.87, and a KMO value of 0.93, suggesting good reliability and validity.^[24]^

### 2.5. Data collection

This study collected data face-to-face from August 2024 to February 2025 via using convenience sampling. All researchers in this study received unified training in August 2024 and began conducting surveys in various locations within the same month. The research was carried out in 5 communities in Nanning City, Guigang City, and Yulin City. For elderly individuals meeting the inclusion and exclusion criteria, researchers detailed the study’s content and procedures, explained the purpose and use of the questionnaire, and obtained voluntary informed consent signatures from participants before they completed the questionnaire. First, the Chinese version of the FRAIL Scale was used to screen potential participants visited, and only older adults with a score of 1 to 2 were met the criteria of pre-frailty and included. For the pre-frail older adults who passed the screening, further assessments were conducted via using a general demographic information questionnaire and the mindful attention awareness scale. Researchers then carefully checked each questionnaire to ensure completeness and authenticity of responses. Each questionnaire took 5 to 10 minutes to complete and collect.

### 2.6. Statistical analysis

The data statistical analysis was performed using SPSS 26.0 and SPSS Modeler 18.0 software. For measurement data, descriptive analysis was conducted using frequency, mean, median, and interquartile range. Categorical variables were statistically described using n (%). The Kolmogorov–Smirnov (K-S) test was used to perform normality tests on mindfulness levels, and the Q-Q plot (Quanyile-Quantile Plot) was combined to determine that it showed a skewed distribution. Mann–Whitney *U* test and Kruskal–Wallis *H* test were used for univariate analysis. Nine variables with statistically significant differences in the univariate analysis were included as independent variables, a multiple linear stepwise regression model was conducted for multivariate analysis. For collinearity diagnosis, the Variance inflation factor (VIF) was used. The VIF values of all variables were <5, indicating no multicollinearity. Taking the mindfulness level as the target variable, variables with statistical significance in the univariate analysis were included in the CRT decision tree algorithm for model construction. The data was recursively partitioned through a binary tree structure to maximize the purity of child nodes. To avoid the “overfitting” phenomenon during the growth process, this study used pre-pruning technology to control model growth. The maximum depth was set to 4, and the minimum sample sizes of parent nodes and child nodes were 15 and 5, respectively. After model grouping, if the parent nodes and child nodes were smaller than the minimum sample size or the number of layers reached the specified depth, the growth would stop.

## 3. Results

### 3.1. Mindfulness levels in pre-frail community-dwelling older adults

Among the 156 pre-frail elderly individuals, there were 64 males (41%) and 92 females (59%). The age distribution primarily ranged from 60 to 89 years. There were 116 urban elderly individuals (74.0%) and 40 rural elderly individuals (25.6%). Among them, 86 individuals (55.1%) had a BMI within the normal range, while 70 individuals (44.9%) had an abnormal BMI. In terms of monthly income, 67 individuals (42.9%) had an income of ≤ 2000 Yuan, 29 individuals (18.6%) had an income of 2000 to 2999 Yuan, 24 individuals (15.4%) had an income of 3000 to 3999 Yuan, and 36 individuals (23.1%) had an income more than 4000 Yuan. The mindfulness level ranged from 39 to 89, with a median of 72.00 and an interquartile range of 13.75. Results of the Mann–Whitney U test and Kruskal-Wallis H test showed that 9 factors had statistically significant effects on the mindfulness level of community-dwelling pre-frail elderly individuals (*P* < .05), including monthly income, place of residence, BMI, educational level, number of children, presence of medical burden, physical exercise duration, presence of pain, and presence of insomnia (Table 1).

**Table 1:** Univariate analysis of factors influencing mindfulness levels in pre-frail community-dwelling older adults (N = 156).

Variable	Group	n (%)	Mindfulness level		
			M (P25, P75)	Z/H	*P*
Gender	Male	64 (41.00)	72.50 (67.00, 77.00)	−0.69	.945
	Female	92 (59.00)	72.00 (64.00, 79.00)		
Age (yr)	60–74	76 (48.72)	71.50 (67.00, 77.00)	0.49	.785
	75–89	74 (47.44)	72.00 (64.50, 79.0)		
	≥90	6 (3.84)	73.50 (65.25, 80.00)		
BMI	Underweight	14 (9.00)	69.00 (56.50, 75.25)	8.10	.044
	Normal	86 (55.10)	73.00 (65.00, 79.00)		
	Overweight	41 (26.30)	69.00 (63.00, 76.00)		
	Obese	15 (9.60)	78.00 (72.00, 81.00)		
Education level	Illiterate	21 (13.50)	69.00 (58.00, 74.50)	11.79	.019
	Primary School	40 (25.60)	68.00 (60.00, 76.75)		
	Junior High School	41 (26.30)	76.00 (67.50, 79.50)		
	High School/Technical Secondary	37 (23.70)	73.00 (65.00, 76.50)		
	College or Higher	17 (10.90)	74.00 (69.00, 81.00)		
Region	Rural	40 (25.60)	68.00 (57.00, 74.00)	−3.09	.002
	Urban	116 (74.40)	73.00 (66.25, 79.00)		
Monthly income (CNY)	≤2000	67 (42.90)	68.00 (60.00, 74.00)	18.31	<.001
	2000–2999	29 (18.60)	70.00 (64.50, 79.00)		
	3000–3999	24 (15.40)	76.50 (68.50, 80.00)		
	≥4000	36 (23.10)	74.50 (71.50, 80.00)		
Number of children	None	3 (1.90)	59.00 (58.00, –)	7.85	.049
	One	46 (29.50)	72.50 (65.50, 79.00)		
	Two	44 (28.20)	74.00 (65.50, 79.00)		
	Three or more	63 (40.40)	69.00 (63.00, 78.00)		
Medical financial burden	Yes	48 (30.80)	68.50 (57.25, 75.75)	−2.99	.003
	No	108 (69.20)	73.00 (76.00, 79.00)		
Personality	Extroverted	78 (50.00)	72.50 (65.75, 79.00)	0.50	.779
	Introverted	31 (19.90)	73.00 (60.00, 78.00)		
	Neutral	47 (30.10)	71.00 (63.00, 78.00)		
Pain assessment	Yes	103 (66.00)	70.00 (63.00, 77.00)	−2.01	.045
	No	53 (34.00)	74.00 (68.50, 79.50)		
Insomnia	Yes	38 (24.40)	71.00 (63.00, 77.00)	−2.25	.024
	No	118 (75.60)	76.00 (68.75, 80.00)		
Daily exercise time	<30min	47 (30.10)	69.00 (60.00, 76.00)	−2.58	.010
	≥30min	109 (69.90)	73.00 (66.50, 79.00)		

H = Kruskal–Wallis *H* Statistic, *P *= *P*-value, Z = Mann–Whitney *U* test Statistic.

### 3.2. Multiple linear regression analysis

Taking the mindfulness level score of community-dwelling pre-frail elderly as the dependent variable, variables with statistical significance in the univariate analysis (monthly income, place of residence, BMI, educational level, number of children, presence of medical burden, and physical exercise duration) were included in the multiple linear regression analysis. The specific variable assignments are shown in Table 2. To avoid interference between variables affecting model accuracy due to the large number of included variables, multiple linear stepwise regression analysis was used. Multivariate analysis results showed that monthly income, presence of medical burden, place of residence, and presence of insomnia were statistically significant (*P* < .05). According to the standardized regression coefficients (Beta), monthly income had the most significant impact on the mindfulness level of pre-frail elderly, followed by place of residence, presence of medical burden, and presence of insomnia. All VIFs were < 5, indicating no multicollinearity issues (Table 3).

**Table 2:** Variables and Assignments.

Variable	Method of assignment
BMI	Underweight=1, normal=2, overweight=3, obese=4
Education level	Illiterate=1, primary school=2, junior high school=3, high school/technical secondary=4, college or higher=5
Region	Rural=1, urban=2
Monthly income (CNY)	≤2000=1, 2000–2999=2, 3000–3999=3, ≥4000=4
Number of children	None=1, one=2, two=3, three or more=4
Medical financial burden	Yes=1, no=2
Pain assessment	Yes=1, no=2
Insomnia	Yes=1, no=2
Daily exercise time	≥30min=1, <30min=2

BMI = body mass index, CNY = Chinese Yuan.

**Table 3:** Multiple linear regression analysis of factors influencing mindfulness levels in pre-frail community-dwelling older adults.

Item	*B*	SE	Beta	*t*	*P*	95% CI	Tolerance	VIF
Constant	47.280	4.333	–	10.912	<.001	38.719–55.840	–	–
Monthly income (CNY)	1.679	0.654	0.208	2.565	.011	0.386–2.972	0.792	1.262
Medical financial burden	3.903	1.606	0.184	2.430	.016	0.729–7.076	0.909	1.100
Region	4.632	1.784	0.207	2.597	.010	1.107–8.156	0.823	1.214
Insomnia	3.880	1.689	0.170	2.298	.023	0.544–7.217	0.950	1.052

95% CI = 95% confidence intervals, *B* = unstandardized regression coefficient, SE = standard error, VIF = variance inflation factor.

### 3.3. Decision tree model development and PCOA mindfulness grouping

**Figure 1.**
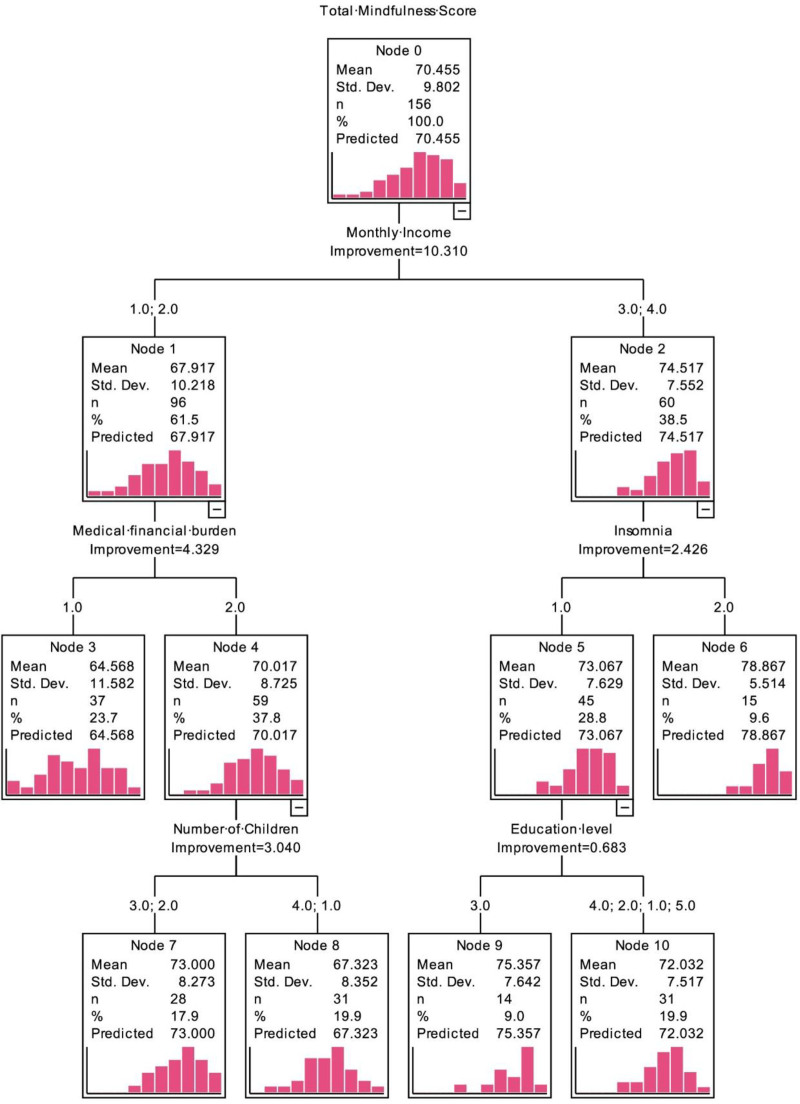
Decision tree model of factors influencing mindfulness levels in pre-frail community older adults.

A risk prediction model for mindfulness levels in community-dwelling pre-frail elderly was constructed based on the CRT decision tree algorithm. The model has 4 layers, 11 nodes and 6 terminal nodes. Five factors including monthly income, medical burden, presence of insomnia, number of children and educational level were selected as explanatory variables, among which monthly income was used as the root node, indicating the strongest correlation with mindfulness level (*F* = 18.645, *P* < .001) (Fig. 1). In the 3-layer node variables, the first layer is monthly income, the second layer is medical burden and presence of insomnia, and the third layer is number of children and educational level, finally generating 6 groups of mindfulness level classifications. The Kruskal-Wallis H test showed good heterogeneity between groups (*P* < .05), and the coefficient of variation (CV) of each group was <1, indicating strong homogeneity within groups and reasonable grouping of mindfulness levels. The PCOA-1 group had the largest number of elderly people (37 cases), the PCOA-4 group had the smallest number (14 cases), the PCOA-6 group had the highest mean mindfulness level (78.87 points), and the PCOA-1 group had the lowest mean mindfulness level (64.57 points) (Table 4).

**Table 4:** Validation of mindfulness level subgroups via principal coordinate analysis based on decision tree classification.

Group	Combination	Number	Proportion (%)	Average mindfulness level	Standard deviation	CV
PCOA-1	Monthly income < 3000 CNY, with medical burden	37	23.70	64.57	11.58	0.18
PCOA-2	Monthly income < 3000 CNY, no medical burden, 1 or 2 children	28	17.90	73.00	8.270	0.11
PCOA-3	Monthly income < 3000 CNY, no medical burden, no children or 3 or more children	31	19.90	67.32	8.352	0.12
PCOA-4	Monthly income ≥ 3000 CNY, with insomnia, education level junior high school	14	9.00	75.36	7.642	0.10
PCOA-5	Monthly income ≥ 3000 CNY, with insomnia, education level illiterate or elementary school or high school or middle school or college and above	31	19.90	72.03	7.51	0.10
PCOA-6	Monthly income ≥ 3000 CNY, no insomnia	15	9.60	78.87	5.514	0.06

CV = coefficient of variation, PCOA = prefrail community-dwelling older adults.

## 4. Discussion

### 4.1. Consistent findings on core influencing factors

Both multiple linear regression (β = 0.208, *P* = .011) and the decision tree model (root node *F* = 18.645, *P* < .001) identified monthly income as the primary factor influencing mindfulness levels, consistent with Gaden research^[24]^ highlighting the significant association between economic status and mental health in older adults. Potential mechanisms include differential access to mindfulness resources (e.g., meditation courses, health exercises) among higher-income groups,^[25,26]^ stress buffering effects of economic security that free cognitive resources for self-awareness development,^[27,28]^ and mediation via health behaviors, as evidenced by the decision tree model showing 82% of individuals with monthly incomes more than 3000 yuan (PCOA-5/6 groups) engaged in more than 30 minutes of daily exercise, which regulates the HPA axis to reduce cortisol and support mindfulness.^[29]^

### 4.2. Complementary analysis of action pathways

The 2 methods revealed the mechanisms of influencing factors from different dimensions. Linear regression quantified the independent effects: mindfulness levels in urban residents were significantly higher than those in rural residents (β = 0.207, *P* = .010), possibly due to the sound support system in urban communities. Medical burden reduced mindfulness levels by 3.9 points (β = 0.184, *P* = .016), confirming the theory that “financial stress related to illness consumes psychological resources.”^[30]^ Insomnia caused a 3.88-point decline in mindfulness levels (β = 0.170, *P* = .023), consistent with neuroscientific findings that sleep deprivation impairs prefrontal function.^[31]^ The decision tree revealed interaction effects: the low-income group with medical burdens (PCOA-1) had the lowest mindfulness level (64.57 points), reflecting the synergistic negative effect of economic constraints and health problems. The buffering effect of intergenerational support was shown as the low-income group without burdens and with fewer children (PCOA-2) had a significantly higher mindfulness level (73 points) than the multi-child group (PCOA-3, 67.32 points), suggesting that families with fewer children may receive more concentrated care support. Educational resources had limited effects on mental health: among high-income insomniacs, the junior high school education group (PCOA-4) had a mindfulness level of 75.36 points, only slightly higher than other educational level groups (72.03 points), indicating that basic education has limited promotion effects on mindfulness levels.

### 4.3. Practical implications of model differences

The decision tree model demonstrates better clinical applicability, with terminal node CV values (0.06–0.18) indicating good within-group homogeneity. In particular, the PCOA-6 group (CV = 0.06) can serve as a “gold standard” reference for community psychological interventions. The regression model holds preventive value by identifying modifiable factors such as insomnia (β=–3.88)^[32]^ providing entry points for early intervention. These findings highlight methodological complementarity: the decision tree captures important interaction terms (e.g., educational level and number of children) omitted by linear regression. Although educational level was included as a classification node in the decision tree model, its improvement value was only 0.683. This weak contribution precisely provides important insights for community interventions: Precisely because of its limited regulatory effect on mindfulness level, it has expanded the scope of application for interventions. Regardless of the educational level of older adults, community workers can focus on the research-verified core targets, such as economic support, sleep improvement, pain management, etc, and improve their mindfulness level through standardized and easy-to-operate protocols. The number of children did not enter the multiple linear regression model, reflecting its weak independent impact on mindfulness level, which may be because its role is regulated by factors such as economic status. However, the decision tree model revealed its key role under specific conditions: in the group with monthly income below 3000 Yuan and without medical burden (Node 3), the number of children became a significant splitting node. The mindfulness level of the group with 1 to 2 children (PCOA-2 group, 73.00 points) was significantly higher than that of the group with no children or 3 or more children (PCOA-3 group, 67.32 points). This result may confirm the hypothesis of moderation in intergenerational support: Families with fewer children may provide more effective psychological buffers for older adults due to lower caregiving pressure and more concentrated emotional bonds; while families with more children may weaken the support effect due to diffusion of responsibility or intergenerational conflicts. While the regression model quantifies unmeasured factors in the decision tree (e.g., the contribution of place of residence). In the regression model, residence passed the significance test (β = 0.207, *P* = .010), indicating that its independent impact on mindfulness level remains statistically significant after excluding the interference of variables such as monthly income, medical burden, and insomnia. This independence may stem from the direct promotional effect of more sophisticated community support systems in urban areas on mindfulness level, including health service resources and social activities. However, decision trees focus more on interactions between variables rather than the independent impact of individual variables. In this study, the influence of residence may be masked by the interaction effects of other variables. For instance, as the root node, monthly income, through its interaction with medical burden and insomnia, can effectively distinguish subgroups with different mindfulness levels; while the impact of residence may depend on these variables, for example, the advantages of urban areas may only manifest in middle- and low-income groups, but such groups have already been prioritized and segmented by variables like monthly income and medical burden. Thus, its independent classification value is weakened and it was not selected as a node in the decision tree. This dual evidence of independent weight combined with contextual interaction clarifies the fundamental role of urban-rural factors in the overall impact, and also suggests that interventions should balance universal improvement of rural basic resources and priority support for rural low-income groups with medical burden.

### 4.4. Intervention strategies to enhance mindfulness

Based on evidence from dual models, a stepped intervention strategy is proposed: First, target the priority group (PCOA-1: low income and medical burden) by establishing community care centers to provide economic and daily necessities assistance, bundled free, low-cost medical services and psychological interventions, and collaborate with hospitals on pain management (due to their high pain prevalence). Second, focus on key targets: promote “sleep-mindfulness” combined interventions,^[33]^ and develop urban community support models adaptable to rural areas to narrow urban-rural gaps. Finally, leverage advantageous resources by cultivating “mindfulness volunteers” from the PCOA-6 group (high income and no insomnia) to expand intervention coverage through peer support.

## 5. Conclusion

This study revealed that the mindfulness level of community-dwelling pre-frail elderly is at a moderate level. Multiple linear regression and CRT decision tree models jointly verified that monthly income is the primary driving factor, and identified medical burden, insomnia, and urban-rural residence differences as key predictive variables, constructing an intervention classification model with 6 homogeneous subgroups. However, the study has limitations: First, this study did not assess the impact of psychological burdens on mindfulness levels, mainly because the use of multiple scales in a cross-sectional design might increase the burden on elderly participants. Second, the convenience sampling was limited to samples from 5 communities across 3 cities, so caution should be exercised when extrapolating the results. Third, the cross-sectional design failed to verify causal pathways. Future research should conduct multicenter, large-sample cohort follow-ups, and further explore through longitudinal tracking or the use of simplified assessment tools, such as the ultra-short version PHQ-2. It should integrate mixed methodologies to enhance the verification of the economic-sleep-intergenerational interaction mechanism and create digital mindfulness tools suited for rural settings, thereby improving the universality and long-term effectiveness of hierarchical interventions.

## Author contributions

**Conceptualization:** Hao Zheng, Junrong Xu.

**Data curation:** Hao Zheng, Fei Li, Yi Qin.

**Formal analysis:** Junrong Xu, Fei Li.

**Funding acquisition:** Yi Qin.

**Supervision:** Yi Qin.

**Visualization:** Hao Zheng, Daoxun Zheng.

**Writing – original draft:** Hao Zheng, Junrong Xu, Daoxun Zheng.

**Writing – review & editing:** Yi Qin.
